# Commercial Bone Grafts Claimed as an Alternative to Autografts: Current Trends for Clinical Applications in Orthopaedics

**DOI:** 10.3390/ma14123290

**Published:** 2021-06-14

**Authors:** Marco Govoni, Leonardo Vivarelli, Alessandro Mazzotta, Cesare Stagni, Alessandra Maso, Dante Dallari

**Affiliations:** 1 Reconstructive Orthopaedic Surgery and Innovative Techniques-Musculoskeletal Tissue Bank, IRCCS Istituto Ortopedico Rizzoli, 40136 Bologna, Italy; alessandro.mazzotta@ior.it (A.M.); cesare.stagni@ior.it (C.S.); dante.dallari@ior.it (D.D.); 2 Laboratory of Microbiology and GMP Quality Control, IRCCS Istituto Ortopedico Rizzoli, 40136 Bologna, Italy; alessandra.maso@ior.it

**Keywords:** autograft alternatives, commercial bone allografts, cellular bone matrices, growth factors, bioactive peptides, xeno-hybrid bone grafts

## Abstract

In the last twenty years, due to an increasing medical and market demand for orthopaedic implants, several grafting options have been developed. However, when alternative bone augmentation materials mimicking autografts are searched on the market, commercially available products may be grouped into three main categories: cellular bone matrices, growth factor enhanced bone grafts, and peptide enhanced xeno-hybrid bone grafts. Firstly, to obtain data for this review, the search engines Google and Bing were employed to acquire information from reports or website portfolios of important competitors in the global bone graft market. Secondly, bibliographic databases such as Medline/PubMed, Web of Science, and Scopus were also employed to analyse data from preclinical/clinical studies performed to evaluate the safety and efficacy of each product released on the market. Here, we discuss several products in terms of osteogenic/osteoinductive/osteoconductive properties, safety, efficacy, and side effects, as well as regulatory issues and costs. Although both positive and negative results were reported in clinical applications for each class of products, to date, peptide enhanced xeno-hybrid bone grafts may represent the best choice in terms of risk/benefit ratio. Nevertheless, more prospective and controlled studies are needed before approval for routine clinical use.

## 1. Introduction

Many clinical conditions, such as arthritis, tumours, traumas, infections (e.g., osteomyelitis, periodontitis), and teeth extraction, may result in bone loss requiring a surgical intervention to replace or restore the lost tissue [1,2]. Furthermore, the ageing population, poised to become one of the most significant social transformations, explains the increasing number of bone graft procedures performed every year.

Musculoskeletal tissue, which includes bone tissue, is a complex system mainly supporting body shape, structure, and locomotion. Over the years, several clinical procedures and material options were investigated for defect repair and bone regeneration [3,4]. Nevertheless, to date, autologous bone is still considered the ideal source for graft procedures since it provides all the three elements necessary for bone healing: (i) an osteoconductive scaffold for cell attachment promotion, (ii) extracellular growth factors for cell proliferation and differentiation, and (iii) viable cells with osteogenic potential [5]. 

However, the clinical application of autologous tissues is limited because of procurement morbidity and constraints on obtainable quantities. These shortcomings are often overcome by advantageous alternative grafts derived from allogeneic or animal sources, albeit these natural replacement materials, compared to autogenous tissues, provide lowered osteoinductive and osteoconductive properties with no osteogenic activity [6]. In this regard, many researchers focused their efforts to combine human or animal bone-derived grafts with specific biological (e.g., growth factors [7], platelet-rich plasma [8], collagen [9]) or synthetic agents (e.g., as calcium sulphate [10], tri-calcium phosphate ceramics [11], bioactive glasses [12], or polymer-based substitutes [13,14]) to enhance the process of osseointegration and bone neoformation in the host. 

Although the scientific literature abounds with promising studies aiming at reconstruction and regeneration, only a few orthopaedic products mimicking autografts reached the market [15]. 

Among these, cellular bone matrices (CBMs) are a class of products that combines osteoinductive and osteoconductive properties provided by nonstructural allografts with the osteogenic potential of viable cells [16]. Each product is made using proprietary techniques and differs in cell type, donor age and gender, and cryopreservation media [17]. Therefore, characteristics related to immunogenicity, cytotoxicity, vascularisation, ability to deliver inductive factors, and cells can potentially endure a variation between different products and within batches of the same grafts. 

Another class of commercial products intended as an alternative to autologous grafts is represented by the growth factor enhanced bone grafts, a combination of natural or synthetic scaffold materials and growth factors produced with recombinant gene technology [18]. In contrast with CBMs, these products exploit growth factors’ signalling activity inducing host multipotent mesenchymal stem cells (MSCs) to become osteoblasts.

Similarly, a recent class of xeno-hybrid bone grafts does not use viable cells to provide osteogenic activity but biomimetic peptides to activate pre-programmed cells to differentiate into competent cells [19]. Since the clinical use of CBMs and growth factors has been associated with significant issues, such as high costs, regulatory matters, or severe complications, the use of bio-active peptides may have the potential to overcome these problems and provide a safe and cost-effective bone grafting option.

The purpose of this review is to describe commercial grafts, which are claimed to be a valid alternative to autologous tissue in terms of osteogenic, osteoinductive, and osteoconductive properties. To this aim, a comprehensive characterisation of each product is performed, considering the manufacturers’ declared properties and critical factors emerging from the scientific literature, which may entail benefits as well as potential shortcomings. Thus, among the complex landscape of current grafting options in orthopaedics, this review aims also at providing an easy and helpful tool to guide clinicians in selecting the products which best fit the clinical indications and relative expected outcomes. 

## 2. Materials and Methods

This review was performed by evaluating only the commercially available products claimed as an alternative to autografts for orthopaedic indications. 

**Figure 1:**
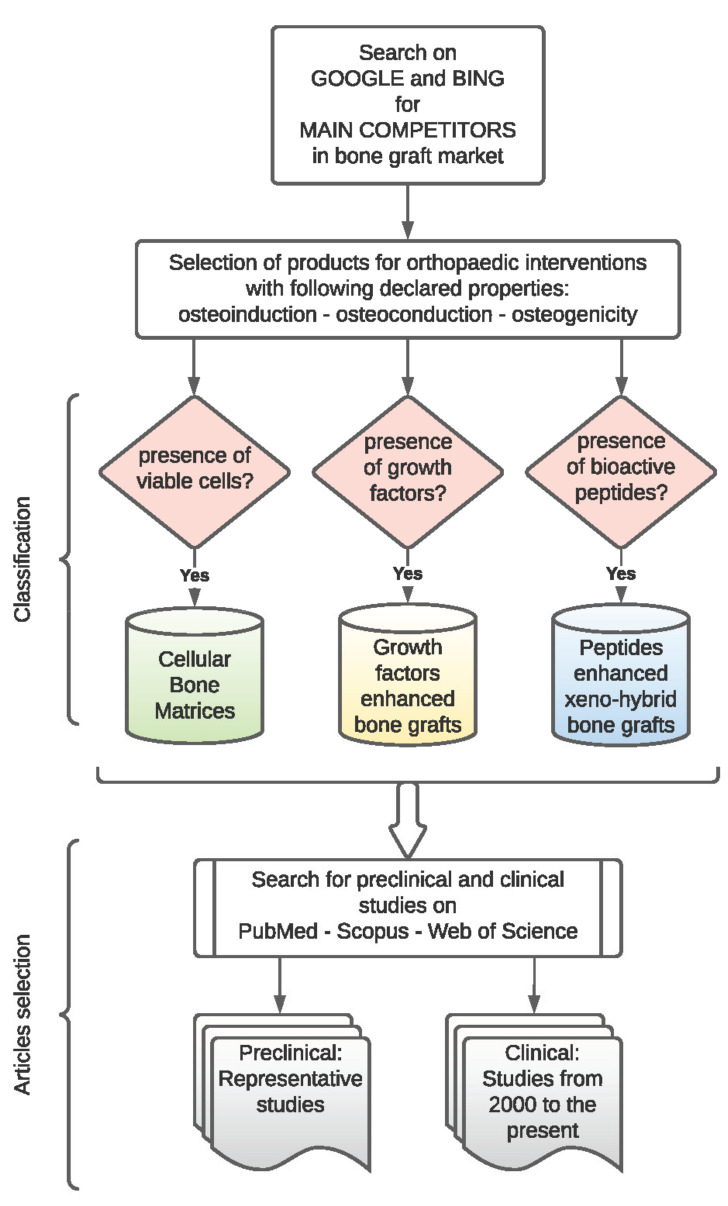
Flowchart showing product classification and study selection.

The search was performed by employing Google and Bing to acquire information from reports or website portfolios of important competitors in the global bone graft market. Only products used for orthopaedic indications and featured osteoinductive/osteoconductive/osteogenic properties were selected. Using this strategy, in the last twenty years, about twenty distinct commercial products were identified on the global market. Successively, based on their characteristics (i.e., presence of cells, growth factors, or bioactive peptides), they were divided into three main categories as reported in Figure 1 and Table 1, Table 2 and Table 3: cellular bone matrices, growth-factor-enhanced bone grafts, and peptide enhanced xeno-hybrid bone grafts. 

The bibliographic databases Medline/PubMed, Web of Science, and Scopus were employed to acquire data and information from preclinical and clinical studies performed to evaluate the safety and efficacy of each product released on the market.

The following term combinations were searched: “commercial name” and “allograft”, “commercial name” and “cellular bone matrices”, “commercial name” and/or “specific growth factor”, “specific growth factor” and “allograft”, “commercial name” and/or “specific peptide”, or “specific peptide” and “xenograft”. Only some representative preclinical studies were discussed to evaluate the safety and efficacy of the selected products, both in vitro or animal models. Regarding clinical studies, the searches were filtered with published data from 2000 to the present, considering I–III Level of Evidence [20]. Only articles written in English were selected. 

The list of website links of these products and the related selected clinical studies discussed in the text are provided in the Supplementary Materials, such as Table S1.

## 3. Results

As listed in Table 1, seventeen cellular bone matrices were identified, although some of them, such as Osteocel^®^ Plus and Osteocel^®^ PRO (Nuvasive, San Diego, CA, USA), Trinity EVOLUTION^®^ and Trinity ELITE^®^ (Orthofix Medical Inc., Lewisville, TX, USA), Via^®^ Graft and Via^®^ Form (Vivex Biologics Inc., Miami, FL, USA), ViviGen^®^ and ViviGen^®^ Formable (DePuy Synthes, Raynham, MA, USA), V92^™^ and V92-FC ^™^ (Paragon 28, Englewood, CO, USA), and SCYLLA^™^ and SCYLLA^™^-F (Chamber Spine. King of Prussia, PA, USA), only differ for composition or formulation. Furthermore, four growth-factor-enhanced bone grafts and two peptide-enhanced xeno-hybrid bone grafts were found, as reported in Table 2 and Table 3, respectively.

### 3.1. Cellular Bone Matrices

Cellular bone matrices (CBMs) or cellular allografts are obtained by proprietary processing techniques that remove all immune-responsive signals generated by bone marrow components. Such components are hematopoietic cells, retaining bone-forming cells within the cancellous bone matrix. However, apart from this feature common to all products, CBMs differ in some critical variables, such as cell type, cell amount, cell viability after thawing, bone tissue processing and subsequent formulation, and cryoprotectant agents. Regarding clinical indications, all products are primarily used as bone filling in surgical treatments of musculoskeletal defects.

#### 3.1.1. Cell Type, Cell Amount, and Post-Thaw Cell Viability 

Viable cells contained within CBMs are cell populations capable of promoting the synthesis of new bone, such as multipotent adult progenitor cells (MAPC), mesenchymal stem cells (MSCs), osteoprogenitor cells (OPCs), or osteoblasts (OBs). MAPCs and MSCs are both non-haematopoietic cells found in bone marrow stroma. These cells retain the ability to self-replicate and differentiate into a specific phenotype by intrinsic and local environmental cues (spatial organisation, mechanical forces, growth factors) [21]. MAPCs are perceived to be a more biologically primitive population and appear to have a greater propensity towards endothelial differentiation than classical MSCs [22]. Besides, both cell populations do not express Class II and co-stimulatory antigens, avoiding immune system recognition and T-cell activation [23]. However, since several authors demonstrated that MAPCs and MSCs elicit humoral and cellular host immune responses, Ankrum et al. suggested considering them not as immune-privileged but instead as immune evasive [24]. Nevertheless, immunomodulatory factors secreted by these cells were shown to suppress the host immune response. Consequently, the true therapeutic effect of these undifferentiated cells relies on their paracrine and autocrine capabilities rather than their innate characteristic of multipotency

The OPCs are located on the endosteal and periosteal surface of the bone and the inner surface of the Haversian canals. They share several features with stem cells, such as differentiative potential and low immunogenicity [25].

The OBs are differentiated mononucleate cuboid cells that are responsible for bone formation. When OBs cease to create new bone, they can become trapped within the matrix and terminally differentiate into osteocytes (OCs; i.e., the most abundant cell type of adult bone tissue [26]). Cryopreservation contributes to reducing the immunogenic potential of allogeneic lineage-bone committed cells [27].

Concerning cell type, cell amount, post-thaw cell viability, and cryoprotectant agents, these characteristics may also be considered as interconnected variables. In this regard, products that contain MSCs and OPCs generally show a high range of cell amounts—between 250,000 and 750,000—especially when dimethyl sulfoxide (DMSO) is used for cryopreservation. On the other hand, in DMSO-free products, this range decreases to 150,000, and post-thaw cell viability percentage exceeds 80%.

Map3^™^ (RTI Surgical, Alachua, FL, USA) utilises only MAPCs as the osteogenic viable cell source. Although the cell count decreases to 50,000 per cc, no information regarding cell viability percentage and cryoprotectant agent is provided.

Up to the present, ViviGen^®^ is the only cellular allograft focused on committed-bone cells instead of the broad-spectrum MSCs: it contains the lowest number of cells per cc (i.e., >16,000/cc) and cell viability exceeds 96%.

#### 3.1.2. Bone Tissue Processing, Components, and Formulations

Osteoconductive and osteoinductive properties of CBMs derive from the use of donated bone tissue that each company processes by its proprietary approach. Specifically, cortical–cancellous bone is machined and transformed in particulate, microparticulate chips or fibres to guarantee essential parameters for maintaining osteoconductive architecture, such as surface area and porosity [28].

Besides, cortical bone particulate or fibers are generally processed through an acid extraction procedure to remove the mineral matrix (i.e., demineralised bone matrix, DBM) and to enhance the bioavailability of collagen and growth factors, such as bone morphogenetic proteins (BMPs), insulin growth factor (IGF), transforming growth factor (TGF), or fibroblast growth factor (FGF), which provide osteoinductive capabilities [29].

So far, BIO^4™^ (Stryker, Kalamazoo, MI, USA) is the only cellular allograft that contains naturally occurring angiogenic growth factors, such as the vascular endothelial growth factor (VEGF), platelet-derived growth factor (PDGF), and basic fibroblast growth factor (bFGF).

CBMs are available in different formats and packaging, although they are commonly provided in three main formulations: particulate, microparticulate, or putty/paste. Differently, Map3^™^ is processed as chips or moldable strips with flexible yet cohesive properties.

Some CBMs, such as V92-FC^™^, SCYLLA^™^-F, and Magnus (Royal Biologics, Hackensack, NJ, USA), use a not well-specified bone gel mixture to obtain a moldable paste with hydrophobic properties that make the graft more lavage resistant.

Interestingly, in V92^™^, V92-FC^™^, Magnus, and CeLLogix (Omnia Medical, Morgantown, WV, USA), each component is provided separately (e.g., cell vial, microparticulate jar, bone gel jar) and needs to be mixed before using.

#### 3.1.3. Bone Cryoprotectant Agents

Cryoprotectant agents were developed to maintain cell viability at extremely low temperatures for long-term storage and transport. They prevent cells from shrinking too quickly and thwart the formation of intracellular ice. Thus, in the case of CBMs, cryopreservation has the purpose of ensuring the osteogenic potential of allogeneic cells providing benefits for the bone grafting site [30].

Among cryoprotectants, dimethyl sulfoxide (DMSO) is widely used because it provides good cell viability after thawing, although its intrinsic cytotoxicity requires rapid removal from grafts before implants [31,32].

However, when DMSO is incorporated with the cells into cortical–cancellous bone components, as it happens for Osteocel^®^ Plus, Osteocel^®^ PRO, Trinity EVOLUTION^®^, Trinity ELITE^®^, or PrimaGen^®^, the removal procedure requires rinsing and decanting steps with 5% dextrose in lactated Ringer’s solution or sterile saline.

Otherwise, more recent CBMs aim at providing a minimal amount of DMSO cryoprotectant (e.g., BIO^4™^) or proprietary DMSO-free cryoprotectant agents (e.g., Via^®^, V92-FC^™^, SCYLLA^™^-F, Magnus, CeLLogix), which make allograft preparation easier since rinsing and decanting steps are not necessary.

### 3.2. Growth Factor Enhanced Bone Grafts

Growth factors are soluble signalling proteins that induce specific biological responses, such as chemotaxis, proliferation, differentiation, anti-apoptotic effects, extracellular matrix synthesis, and angiogenesis [33,34].

Promising preclinical and clinical results may lead to the subsequent introduction of various recombinant human growth factors into the commercial market. However, so far, only two genetically engineered proteins are used within commercially claimed autograft replacements to regulate bone healing and growth (Table 2). Such proteins are recombinant bone morphogenic protein 2 (rhBMP-2) and recombinant human platelet-derived growth factor-BB homodimer (rhPDGF-BB).

Bone morphogenic proteins (BMPs) are a soluble member of the transforming growth factor-beta (TGF-β) superfamily, involved in the osteoinduction process and the resulting endochondral ossification [35].

With the scientific advances in genetic cloning, it was possible to produce large quantities of BMPs for clinical use. Mainly, rhBMP-2, produced by a genetically engineered Chinese hamster ovary cell line, represents the active agent in INFUSE^®^ (Medtronic Spinal and Biologics, Memphis, TN, USA), one of the most used products in spinal fusion procedures.

As a carrier for the delivery of rhBMP-2, INFUSE^®^ exploits an absorbable collagen sponge (ACS) made from bovine Type I collagen obtained from the deep flexor tendon. ASC is a soft and pliable matrix that also acts as a scaffold for new bone formation. However, due to the lack of mechanical support, it should not be used to fill space in the presence of compressive forces.

PDGF-BB is a potent chemo-attractant and mitogen factor for cells involved in wound healing, including MSCs, OCs, and tenocytes [36]. In addition, PDGF-BB plays a pivotal role in blood vessel formation and angiogenesis upregulation. An engineered version of this native protein is firstly provided by Gem 21S^®^ (Lynch Biologics, Franklin, TN, USA) and then by Augment^®^ (Wright Medical Group N.V., Memphis, TN, USA), growth-factor-enhanced bone grafts that combine the osteoinductive capabilities of rhPDGF-BB with the osteoconductive properties of a bioresorbable synthetic scaffold, namely beta-tricalcium phosphate (β-TCP). Specifically, β-TCP facilitates the delivery of the added growth factor and prevents soft tissue from collapsing into the void.

OsteoAMP^®^ (Bioventus LLC, Durham, NC, USA) may be considered as an exception among this class of products since its peculiarity is represented by a proprietary processing procedure designed to retain essential endogenous growth factors from allogeneic iliac crests. This unique production process allows obtaining a sort of allogeneic morphogenic protein (AMP) that retains up to 23 different growth factors, such as BMP-2, BMP-7, TGF-β1, aFGF, VEGF, and angiopoietin 1 (ANG1). Therefore, no recombinant growth factors or carriers are added to formulations. Furthermore, when OsteoAMP^®^ is provided as granules with the addition of mineralised cortical-cancellous allograft chips, it confers load-bearing structural support.

### 3.3. Peptide Enhanced Xeno-Hybrid Bone Grafts

Different strategies were implemented to improve bone regeneration using a different combination of bone sources, biomaterials, or biomolecules. Recent trends point towards a composite approach for best mimicking the human bone structure [37]. In this regard, xeno-hybrid bone grafts that combine osteoconductive properties provided by animal origin bone matrix with osteoinductive/osteogenic capabilities derived from bioactive peptides, resulting particularly efficient for bone tissue regenerative purposes. However, at present, we can only find a limited amount of these innovative products (Table 3).

Arginyl-glycyl-aspartic acid (RGD) sequence is found in several extracellular matrix (ECM) molecules, such as fibronectin and collagen. It is well-established that RGD peptides enhance cell attachment and the spreading of OBs onto graft materials. Peptides also increase cellular proliferation and promote OB differentiation and mineralisation [38].

SmartBone^®^ (IBI, Mezzo-Vico Vira, Switzerland) is a bone substitute composed of bovine bone matrix, micrometric thin poly(l-lactic-co-*ε*-caprolactone), and RGD-containing bovine collagen fragments, used for dental and orthopaedic indications. SmartBone^®^ is provided in several formulations, such as microchips, blocks, plates, wedges, or custom-made bone grafts specifically designed on the patient’s defect.

The P-15 peptide is a highly conserved peptide that consists of a 15 amino acids linear sequence (GTPGPQGIAGQRGVV) identical to the cell-binding region of collagen type I. The P-15 is immobilised on a bovine anorganic bone matrix, suspended within an inert, biocompatible hydrogel, which represents an ideal scaffold for bone growth [39].

This patented synthetic non-RGD protein segment, originally contained within PepGen P-15 (DENTSPLY Friadent CeraMed, Lakewood, CO, USA), today within i-FACTOR^®^ (Cerapedics, Westminster, CO, USA), provides a novel mechanism of action based on the cell binding of OPCs via integrins, or signal receptors, which promote cell attachment to bone substitutes and upregulates extracellular matrix production. Specifically, once cells attach to the P-15, the signalling pathways are activated, and the cascade of events leading to new bone formation commences. Regarding formulations, i-FACTOR^®^ is provided as putty and available worldwide. i-FACTOR can also be provided in the form of flexible strips following a freeze-drying treatment, not commercially available in the USA.

## 4. Discussion

Bone grafting surgical interventions performed worldwide per year exceed two million procedures [40]. Therefore, although autografts are still considered the best option for hard tissue repair, autografting cannot meet the overall medical demand for orthopaedic implants. Nowadays, many bone graft alternatives are available for clinical use, following the evolution of biomaterials, implant designs, and innovative processing techniques. However, effective reconstructive treatments remain challenging, especially considering that each bone substitute has advantages and disadvantages [41]. Furthermore, although the market offers a wide variety of products for clinical use, this range is significantly reduced to three main categories of products when searching for alternative bone augmentation materials mimicking autografts: (i) cellular bone matrices (CBMs), (ii) growth factor enhanced bone grafts, and (iii) peptide enhanced xeno-hybrid bone grafts.

Advances in stem cell technology and the innate capability of allogeneic bone tissue to allow a uniform loading and retention of MSCs, rapid vascular ingrowth, and incorporation into the bone host, focused the attention on the development of cellular allografts showing all three elements necessary for bone growth and healing: osteoinduction, osteoconduction, and osteogenic activity.

Such products are manufactured in the USA, where they are regulated by the less scrupulous section 361 of the Public Health Service Act and Code of Federal Regulations (CFR) title 21 section 1271, which does not require Food and Drug Administration (FDA) premarket review and approval [16,42,43]. However, this approach should not mislead the assumption that CBMs are not continuously supervised. Indeed, so far, there have been injunctions issued by the FDA against several companies for providing products that do not satisfy the following criteria: minimal manipulation; homologous use only; systemic effect absence; the primary function not dependent on the metabolic activity of viable cells, unless the product is intended for autologous use or use by a first- or second-degree blood relative. If a product fails to meet at least one of these criteria, it must be regulated as a drug, device, or biological product and requires a lengthy premarket review [44]. This is the case of Map3^®^ cellular allograft, which was removed from the market in 2018 by RTI Surgical after losing a four-year battle with the FDA [43]. Another similar case was the one of Ovation^®,^ which was warned after an FDA inspection of Osiris Therapeutics (Columbia, MD, USA) in early 2013 (Table 4) [45].

**Table 1:** Summary of cellular bone matrices commercially available for use.

Commercial Name/Manufacturer	Cell Type/Amount/Viability Post-Thaw	Composition	Formulations	Cryoprotectant Agent	Clinical Indications	Clinical Studies [Ref.] or NCT
Osteocel^®^ Plus Osteocel^®^ PRO Nuvasive, San Diego, CA, USA	MSCs and osteoprogenitor cells. >250,000 cells/cc > 70%	Cryopreserved viable cancellous matrix. Ground demineralised bone matrix.	Particulate Putty	DMSO	Spine, orthopaedics, oral and maxillofacial applications.	[46,47,48,49,50,51]
Trinity EVOLUTION^®^ Trinity ELITE^®^ Orthofix Medical Inc., Lewisville, TX, USA	MSCs and osteoprogenitor cells. >750,000 cells/cc ≥70%	Cancellous bone. Demineralised bone particulates or Cancellous bone. Demineralised bone fibres.	Putty	DMSO	Treatment of musculoskeletal defects.	[52,53,54,55]
Via^®^ Vivex Biologics Inc., Miami, FL, USA	Bone-derived cells. >150,000 cells/cc >80%	100–300 µM demineralised cortical bone. Mineralised cortical and cancellous bone (Via^®^ Graft). Cortical shavings, crushed cancellous chips (Via^®^ Form).	Particulate Paste	ViaCoat™ DMSO-free cryoprotectant	Spine, upper extremity, foot and ankle, oral and maxillofacial, and orthopedic oncology.	[56]
ViviGen^®^ ViviGen^®^Formable DePuy Synthes, Raynham, MA, USA	Osteoblasts, osteocytes, and bone lining cells. >16,000 cells/cc ≥96%	Corticocancellous chips. Demineralised bone particulate or fibres.	Particulate Putty	Proprietary cryopreservation medium	Fusion, non-union, and malunion for foot/ankle, long bone, and craniomaxillofacial trauma and reconstruction in patients with compromised biology.	[57,58,59,60]
BIO^4™^ Stryker, Kalamazoo, MI, USA	MSCs osteoprogenitor and osteoblasts. >600,000 cells/cc >70%	Native matrix. Endogenous Osteoinductive and angiogenic growth factors.	Putty	Minimal amount of a proprietary cryopreservation medium	Treatment of musculoskeletal defects.	NCT03077204
PrimaGen^®^ Zimmer Biomet, Warsaw, IN, USA	MSCs, osteoprogenitorcells, pre-osteoblasts >750,000 cells/cc >70%	Cancellous bone. Demineralised cortical bone.	Putty	N/A	Treatment of musculoskeletal defects.	NCT02182843
Map3^®^ RTI Surgical, Alachua, FL, USA	MAPC-class cells. >50,000 cells/cc n.a.	Cortical cancellous bone chips. Demineralised bone matrix.	Strips Chips	N/A	Small joint repair, filling bone defects.	n.a.
V92^™^ V92-FC ^™^ 28, Englewood, CO, USA	Bone-derived cells. >150,000 cells/cc >80%	Cortical cancellous bone particulate. Demineralisedbone matrix. Bone gel (only in V92-FC^™^)	Microparticulate Paste	DMSO-free cryoprotectant	Orthopaedic and spine applications.	n.a.
SCYLLA^™^ SCYLLA^™^-F Chamber Spine. King of Prussia, PA, USA	Bone-derived cells. >150,000 cells/cc >80%	Cortical cancellous bone particulate. DBM and bone mixture gel (only in SCYLLA^™^-F).	Microparticulate Paste	DMSO-free cryoprotectant	Treatment of musculoskeletal defects.	n.a.
Magnus Royal Biologics, Hackensack, NJ, USA	Cell population with MSC and pluripotent cell markers >150,000 cells/cc >80%	Cortical shavings, crushed cancellous chips, and demineralised cortical bone microparticulate scaffold blend with bone gel mixture.	Paste	DMSO-free cryoprotectant	Fusion, midfoot arthrodesis.	n.a.
CeLLogix Omnia Medical, Morgantown, WV, USA	Bone-derived cells. >150,000 cells/cc >80%	Cortical–cancellous bone particulate. DBM	Microparticulate	DMSO-free cryoprotectant	Treatment of musculoskeletal defects.	n.a.

Osteocel^®^ was introduced to the market about sixteen years ago and represents the first CBM used in clinics. Several studies have shown that it is a safe and effective product for bone healing in several surgical treatments, such as hindfoot and ankle arthrodesis [46], anterior cervical discectomy [47], lumbar or extreme lateral fusion procedures [48,49,50], and maxillary sinus floor augmentation [51].

Likewise, Trinity Evolution^®^, released in 2009 by Orthofix, has demonstrated high fusion rates and no safety-related concerns after the implant. Specifically, prospective clinical studies were performed to assess the radiographic and clinical outcomes of this viable cellular bone allograft in subjects undergoing single- or two-level anterior cervical discectomy and fusion [52,53], and undergoing one- and two-level posterolateral lumbar arthrodesis with decompressive laminectomy [54].

Four years later, Orthofix announced the full market release and launch of Trinity Elite^®^ that differs from Evolution^®^ ‘s formulation for the count of MSCs and/or OPCs that is 2-fold greater (≥100,000/cc vs. ≥50,000/cc cells, respectively). Moreover, the presence of DBM fibres, instead of DBM particulates, makes Trinity Elite^®^ more resistant to irrigation and more deeply packed into bone defects. Recently, Loveland et al. [55] performed a retrospective clinical comparison of these two similar products, showing that both Trinity Evolution^®^ and Elite^®^ effectively achieve comparable fusion rates in patients undergoing foot and/or ankle arthrodesis.

In 2014, the Via^®^ series (Graft or Form) and Vivigen^®^ were launched on the market by Vivax and DePuy Synthes, respectively. A retrospective study on patients treated with Via^®^ Graft for both primary and revision surgery showed a 96% fusion rate at 12 months postoperative follow-up, demonstrating the safety and effectiveness of the cellular allograft used during surgical interventions [56]. However, at present, this is the only published study. On the other hand, several studies reporting good results were presented on the use of Vivigen^®^ in anterior and posterior cervical fusion [57], posterolateral lumbar spine fusion [58], two-stage total hip arthroplasty [59], and ankle arthrodesis [60]. ViviGen^®^ represents a sort of paradigm shift among CBMs since it is the only one focused on committed bone cells instead of the broad-spectrum MSCs. This choice was based on studies that demonstrated that OBs stay at the defective site longer [61] and secrete the chemotactic factor IGF-1 to recruit additional osteoblasts [62].

In 2015, Stryker introduced on the market BIO^4™^, claimed as the next generation of CBMs since it exploits a fourth characteristic involved in bone repair and regeneration: angiogenic activity. This peculiarity derives from the presence of growth factors, such as VEGF, PDGF, and bFGF, kept intact after the non-proteolytic processing of periosteum. However, to the authors’ best knowledge, no clinical studies exist to carry out a retrospective or comparative evaluation of this product. Only a prospective open-label study was launched in 2017 (Identifier: NCT03077204) to evaluate clinical and radiographic outcomes of BIO^4™^ in 20 patients undergoing 1- or 2-level anterior cervical discectomy and fusion surgery. However, at present, no data are yet available. Recently, in 2020, Lin et al. [63] compared the ability of several commercially available CBMs, including BIO^4™^, to form a stable spinal fusion using an animal model of posterolateral fusion. However, the results show that BIO^4™^ failed this aim, although it was possible to observe an increase in total bone volume.

PrimaGen^®^, formerly called Cellentra^®^, was introduced in 2016 by Zimmer Biomet. Apart from a clinical trial, registered on ClinicalTrials.gov (Identifier: NCT02182843), with the purpose to assess the clinical and radiographic outcomes in patients who undergo anterior cervical discectomy and fusion procedures using Cellentra, at present, there is no data to suggest a fusion rate by PrimaGen^®^.

More recently, DMSO-free viable grafts such as V92^™^, Scylla^™^, Magnus, and CeLLogix reached the market. However, the research on Medline/PubMed, Scopus, and Web of Sciences, does not yet show available studies on these products.

Therefore, although for older CBMs retrospective studies show promising results in terms of efficacy and safety, large randomised clinical trials may be required to solidify the role of allograft with viable cells. The need for well-designed clinical studies on cellular graft materials is also emphasised by current studies on animal models that show controversial data [17,63]. Besides, CBM comparative studies on patients may contribute to better understanding, which product represents the best choice for a specific clinical indication and cost-effectiveness ratio. However, for each CBM, it should be taken into account that several intrinsic biological characteristics, such as viable cell sources, the donor age at the time of graft harvest, or cell survival after transplantation, may cause variations among different lots of the same product in terms of expected outcomes.

Another class of products emerged to meet the need for grafting materials capable of circumventing the inherent drawbacks of autologous transplantation is represented by the growth factor enhanced bone grafts, even if they are associated, as well as CBMs, with higher costs compared with other conventional grafts [16,64].

INFUSE^™^ is the first commercially available product that has exploited the advances in genetic engineering and biological technology for bone grafting purposes. From 2002, when INFUSE^™^ was initially approved by the FDA, up to now, it is probably the most researched and published bone graft material. Indeed, rhBMP-2 is an active agent and was extensively studied in several preclinical animal models, including non-human primates [65]. These studies consistently showed rhBMP-2 to be equivalent and, in many cases, superior to autogenous bone. Likewise, the rhBMP-2 fusion rate on patients was usually compared with the autologous iliac crest bone graft (ICBG) both for its ability to form de novo bone as well as clinical outcomes [66,67].

Thus, after the FDA approval, the use of rhBMP-2 dramatically increased in the USA [68], thanks also to initial industry-supported studies that showed no significant side effects in various surgical procedures [69,70,71].

However, many notable complications, such as retrograde ejaculation, seroma formation, heterotopic ossification, osteolysis, neurological deficits, and an increased risk of cancer, started to be observed in patients treated with rhBMP-2 [72]. Moreover, an FDA warning (Table 4) was issued not to use rhBMP-2 in the anterior cervical spine due to inflammation causing severe dysphagia and a potential increase in mortality [73].

At present, INFUSE^™^ is indicated for use in interbody spine fusion, fresh tibial fractures, and oral maxillofacial bone grafting procedures. Moreover, Medtronic has recently announced a new clinical trial for expanding the use of INFUSE^™^ in transforaminal lumbar interbody fusion (TLIF) spine procedures [74].

Nevertheless, the controversy surrounding the use of rhBMP-2 in bone augmentation procedures was not completely addressed. Although it is difficult to determine the ideal candidate for rhBMP-2 enhanced bone grafts, James et al. asserted that their use might be indicated as a second adjunct line to spinal fusion where other alternatives are either not available or not likely to lead to effectiveness [75].

On the other hand, in a recent meta-analysis and systematic review on comparative clinical effectiveness and safety of rhBMP-2 vs. autologous ICBG in lumbar fusion, Liu et al. concluded that there was no difference in the incidence of adverse events between rhBMP and ICBG [76].

In 2005, the FDA approved Gem 21S, the first entirely synthetic product combining a purified recombinant growth factor (rhPDGF-BB) with a synthetic bone matrix (β-TCP) to treat periodontal-related defects. Specifically, rhPDGF-BB provides a biological stimulus for the recruitment and proliferation of cells, including OBs, responsible for the formation of bone, while β-TCP provides mechanical support. About ten years later, the FDA approved AUGMENT^®^, nearly identical to Gem 21S regarding composition but developed for ankle and/or hindfoot fusion indications. These FDA approvals are consequent to preclinical [77,78,79,80] and clinical [81,82,83,84] studies that have shown the safety and efficacy of rhPDGF-BB. However, its use is not without risks. Specifically, as well as for rhBMP-2, the possibility of increased cancer rates for drugs with a cell growth-promoting property should be taken into account since rhPDGF-BB promotes the growth of granulation tissue and wound healing via interaction with receptors on fibroblasts and endothelial cells. Therefore, rhPDGF-BB should be used with caution in patients with known malignancy and only used when the benefits can be expected to outweigh the risks [85].

**Table 2:** Summary of growth factor enhanced bone grafts commercially available for use.

Manufacturer	Commercial Name	Active Molecule	Carrier	Formulations	Clinical Indications	Clinical Studies [Ref.] or NCT
Medtronic Spinal and Biologics, Memphis, TN, USA	INFUSE^®^	rhBMP-2	ACS	Vial + sponge	Spinal fusion procedures. Treatment of open tibial fractures with an intramedullary (IM) nail fixation. Sinus floor and alveolar ridge augmentations.	[66,67,68,69,70,71,72,73,74,75,76,86]
Lynch Biologics, Franklin, TN, USA	Gem 21S^®^	rhPDGF-BB	β-TCP	Vial + granules	Periodontal defects.	n.a.
Wright Medical Group N.V., Memphis, TN, USA	Augment^®^	rhPDGF-BB	β-TCP	Vial + granules	Arthrodesis (i.e., fusion procedures) of the ankle and/or hindfoot.	[81,82,84]
Bioventus LLC, Durham, NC, USA	OsteoAMP^®^	AMP	n.a.	Granules, putty, or sponge	Cervical/lumbar spine fusion.	[86,87] NCT02225444

OsteoAMP^®^ is an innovative bone allograft that was processed to retain multiple endogenous growth factors for use in spinal fusion procedures. Therefore, no recombinant highly purified proteins are added to this product. However, despite the attractive rationale for the use of OsteoAMP^®^, few studies demonstrating its efficacy are available, performed mainly by authors with competing interests, since they declared to be unpaid consultants for Advanced Biologics (i.e., the company that had launched OsteoAMP^®^) and/or hold shares in the company [86,87].

A clinical study was launched in 2015 (Identifier: NCT02225444) to evaluate the efficacy of OsteoAMP^®^, in terms of fusion rates, adverse events, and pain and health scores, in patients requiring instrumented posterolateral spinal fusion procedure of the lumbar or lumbosacral spine, 1 to 2 adjacent levels. However, although this clinical trial was concluded in 2019, at present, no data are yet available. Therefore, further studies to better assess long-term results are needed.

Hence, commercially available growth factor enhanced bone grafts can improve surgical outcomes and represent a valid alternative to autogenous bone transplantation. Nevertheless, long-term effects not clearly identified, off-label use in the paediatric population, and limited use in oncologic patients, are relevant issues that may lead to the demand for new signalling systems. In this regard, the discovery that some peptides have the ability to upregulate bone healing without severe side effects and prohibitive costs may contribute to overcoming some of the abovementioned problems [88].

However, although a significant number of peptides were developed and investigated as potential candidates for bone healing, to date, only a few of them reached the market as active agents.

In 2012, IBI introduced SmartBone^®^ on the EU and international market, an innovative osteoconductive and osteoinductive bone substitute featured by a bovine mineral bone matrix, bioresorbable polymers, and RGD-containing bovine collagen fragments. To date, several studies demonstrated its capabilities to promote osseointegration and cell differentiation in oral, maxillofacial, and cranial surgery [89,90,91,92]. Furthermore, in recent years, IBI has received approval to expand the use of SmartBone^®^ (i.e., SmartBone^®^ ORTHO) also in the orthopaedic field, albeit available clinical studies are still limited and performed mainly by authors with competing interests [93,94]. Therefore, although data show that SmartBone^®^ is a safe biomaterial that induces a high grade of osseointegration and remodelling with satisfactory mechanical performances, further independent clinical studies are needed to confirm these promising results in orthopaedic applications.

In 2014, Cerapedics launched i-FACTOR^®^ on the market, a biologic bone graft featured by the P-15 osteogenic cell-binding peptide bound to an anorganic bovine bone matrix. Investigations on the ability of this product to favouring cellular attachment, and consequent new bone formation, started about 20 years ago, as attested by numerous preclinical studies [95,96,97,98,99,100], and continued by clinical trials [101,102,103,104] that allowed the establishment of its safety and the efficacy to replace or augment autologous bone.

To date, i-FACTOR^®^ is indicated for common orthopaedic applications, such as spinal fusion procedures, treatment of non-union or fresh traumatic fractures, and joint reconstruction. Besides, last year, Arnold et al. showed that diabetes is not a contraindication for patients requiring single-level surgery for cervical degenerative disc disease when i-FACTOR^®^ or local autologous bone is used [105].

**Table 3:** Summary of peptide enhanced xeno-hybrid bone grafts commercially available for use.

Manufacturer	Commercial Name	Peptide	Composition	Formulations	Clinical Indications	Clinical Studies [Ref.] or NCT
IBI, Mezzo-Vico Vira, Switzerland	SmartBone^®^	RGD	Bovine bone matrix. Micrometric thin poly(l-lactic-co-*ε*-caprolactone).	Microchips Blocks, plates, wedges	Dental and orthopaedic indications.	[89,90,91,92,93,94]
Cerapedics, Westminster, CO, USA	i-FACTOR^®^	P-15	Anorganic bovine bone matrix. Inert biocompatible hydrogel.	Putty Strips (not in the USA)	Bone filling defects in the spine and extremities.	[101,102,103,104,105,106]

Compared to SmartBone^®^, the composition of i-FACTOR^®^ lacks a resorbable biopolymer that confers mechanical support. As a consequence, it is not intended to provide load-bearing structural support during the healing process, while rigid fixation techniques are strongly recommended to assure stabilisation of the defect in all planes.

Concerning the use of peptide enhanced bone grafts in the young population, little is recorded with regards to their potential complications. Oxborrow et al. recommend long-term studies to assess the efficacy, safety, and complications associated with the use of i-FACTOR^®^ in children since they documented heterotopic ossification following spinal fusion with this bone graft substitute in an eight-year-old patient affected by mucopolysaccharidosis [106].

Interestingly, the IBI company has declared on its website to be committed to the clinical studies that will soon introduce in the market a new composite bone substitute intended for the regeneration of bone defects and losses in paediatric applications, including oncological ones. This innovative bone graft material, named SmartBonePep^®^ [107], has the same composition as SmartBone^®^ except for the addition of synthetic peptides that reproduce several proline-rich regions present in the intrinsically disordered proteins (IDPs), a protein family involved in biomineralisation [108]. Therefore, considering the limited number of bone grafting options in children, SmartBonePep^®^ may sound like an appealing alternative to autogenous iliac bone graft that is still considered the benchmark of bone transplantation procedures in the paediatric population.

**Table 4:** Summary of withdrawn or warned commercial bone grafts.

Manufacturer	Commercial Name	FDA Injunction	Status
Osiris Therapeutics, Columbia, MD, USA	Ovation^®^	The manufacturing process alters the original relevant characteristics of the tissue. The product is dependent upon the metabolic activity of living cells for their primary function and is not intended for autologous use or allogeneic use in a first- or second-degree relative [45].	Withdrawn. It was transitioned to Ovation OS and currently available as BIO^4™^ (distributed by Stryker).
RTI Surgical, Alachua, FL, USA	Map3^®^	The processing does not meet the definition of minimal manipulation for cells or nonstructural tissues [109].	Withdrawn.
MedtronicSpinal and Biologics, Memphis, TN, USA	INFUSE^®^	FDA warning was issued not to use in the anterior cervical spine due to inflammation causing severe dysphagia and a potential increase in mortality [73].	Available for use in interbody spine fusion, fresh tibial fractures, and oral maxillofacial bone grafting procedures.

## 5. Conclusions

Over the last two decades, researchers and clinicians have striven to achieve technological advances in bone grafting to ameliorate spinal fusion treatments, bony voids, fractures, and post-traumatic non-unions. Commercially available cellular bone matrices and growth factor/peptide enhanced bone grafts are claimed as a valid alternative to the autologous bone for osteogenic/osteoinductive and osteoconductive properties. Nevertheless, an accurate characterisation of each product has shown potential drawbacks that may reduce the emphasis related to these bone substitutes. Furthermore, most of the scientific literature evaluating autologous bone alternatives consists of low-level studies and case series. On the other hand, large randomised clinical trials and prospective cohort studies with high-quality design and execution are mandatory to better enhance the optimal treatment for patients undergoing bone grafting, especially for products that continue to dominate the market.

Taking into account critical issues, mainly related to CBMs and growth factor-based products, such as high costs, regulatory issues, or severe complications, peptide enhanced xeno-hybrid bone grafts may represent the best choice in terms of risk/benefit and cost-effectiveness ratios. Peptides can trigger some specific signalling pathways that control osteogenic-related cellular functions, have low immunogenicity, are easily synthesised and handled, and to date, no severe side effects have been reported.

Nevertheless, at present, bioactive peptides are exclusively used in combination with xenogeneic bone sources. In contrast, based on the biomimetic principle (i.e., a material as similar as possible to the host bone is recommended to allow for the best biological behaviour [6,110]), it could be interesting to investigate the synergistic effect exerted by peptides and allogeneic bone tissues.

Hence, further scientific efforts should be encouraged to promote a translational approach that bridges research and clinical setting, intending to minimise potential biases concerning the efficacy and safety of both innovative and outdated products.

## Data Availability

No new data were created or analysed in this study. Data sharing is not applicable to this article.

## Supplementary Materials

The following is available online at https://www.mdpi.com/article/10.3390/ma14123290/s1, Table S1: List of website links of commercially available products claimed as an alternative to autografts.
