# Supplementary material for: Commercial Bone Grafts Claimed as an Alternative to Autografts: Current Trends for Clinical Applications in Orthopaedics
Source: Materials (Basel). 2021 Jun 14;14(12):3290. doi: 10.3390/ma14123290 (PMC8232314; doi:10.3390/ma14123290)
Supplement: Supplementary file 1 [file materials-14-03290-s001.zip › materials-1257184-supplementary.pdf]

materials

Supplementary Materials

Commercial Bone Grafts Claimed as an Alternative to Autografts: Current Trends for Clinical Applications in Orthopaedics

Marco Govoni [1,*], Leonardo Vivarelli [1,*], Alessandro Mazzotta [1], Cesare Stagni [1], Alessandra Maso [2] and Dante Dallari [1]

[1] Reconstructive Orthopaedic Surgery and Innovative Techniques-Musculoskeletal Tissue Bank, IRCCS Istituto Ortopedico Rizzoli, 40136 Bologna, Italy; alessandro.mazzotta@ior.it (A.M.); cesare.stagni@ior.it (C.S.); dante.dallari@ior.it (D.D.)
[2] Laboratory of Microbiology and GMP Quality Control, IRCCS Istituto Ortopedico Rizzoli, 40136 Bologna, Italy; alessandra.maso@ior.it
* Correspondence: marco.govoni@ior.it (M.G.); leonardo.vivarelli@ior.it (L.V.)

Table S1. List of website links of commercially available products claimed as an alternative to autografts.

Class of Bone Grafts	Manufacturer	Commercial Name	Website Link
**Cellular bone matrices**	Nuvasive, San Diego, CA, USA	Osteocel® Plus<br>Osteocel® PRO	https://www.nuvasive.com/procedures/featured-offerings/biologics/ (accessed date: 09 June 2021)
	Orthofix Medical Inc., Lewisville, TX, USA	Trinity EVOLUTION®	https://www.orthofix.com/ifus/trinity-evolution/ (accessed date: 09 June 2021)
		Trinity ELITE®	https://www.orthofix.com/ifus/trinity-elite/ (accessed date: 09 June 2021)
	Vivex Biologics Inc., Miami, FL, USA	Via® Graft<br>Via® Form	https://vivex.com/by-brand/via/ (accessed date: 09 June 2021)
	DePuy Synthes, Raynham, MA, USA	ViviGen®<br>ViviGen® Formable	https://www.jnjmedicaldevices.com/en-US/product/vivigen-cellular-bone-matrix (accessed date: 09 June 2021)
	Stryker, Kalamazoo, MI, USA	BIO4™	https://www.stryker.com/us/en/trauma-and-extremities/products/bio4.html (accessed date: 09 June 2021)
	Zimmer Biomet, Warsaw, IN, USA	PrimaGen®	https://www.zimmerbiomet.com/medical-professionals/spine/product/primagen-advanced-allograft.html (accessed date: 09 June 2021)
	RTI Surgical, Alachua, FL, USA	Map3®	https://www.lifehealthcare.com.au/wp-content/uploads/2017/06/MAP3-Technical_Brochure_FINAL.pdf (accessed date: 09 June 2021)
	Paragon 28, Englewood, CO, USA	V92™<br>V92-FC™	https://www.paragon28.com/products/v92-cellular-bone-matrix/ (accessed date: 09 June 2021)

	Chamber Spine. King of Prussia, PA, USA	SCYLLA™ SCYLLA™-F	https://www.cambermedtech.com/scylla-cellular-bone-matrix (accessed date: day month year)
	Royal Biologics, Hackensack, NJ, USA	Magnus	https://royalbiologics.com/magnus-graft/ (accessed date: 09 June 2021)
	Omnia Medical, Morgantown, WV, USA	CeLLogix	https://omniamedical.com/products/cellogix (accessed date: 09 June 2021)
**Growth factor enhanced bone grafts**	Medtronic Spinal and Biologics, Memphis, TN, USA	INFUSE®	https://global.medtronic.com/xg-en/e/response/infuse-bone-graft.html(accessed date: 09 June 2021)
	Lynch Biologics, Franklin, TN, USA	Gem 21S	https://www.lynchbiologics.com/products/gem-21s/ (accessed date: 09 June 2021)
	Wright Medical Group N.V., Memphis, TN, USA	Augment®	https://www.augmentbonegraft.com/ (accessed date: 09 June 2021)
	Bioventus LLC, Durham, NC, USA	OsteoAMP®	https://www.bioventussurgical.com/product/osteoamp/ (accessed date: 09 June 2021)
**Peptide enhanced xeno-hybrid bone grafts**	IBI, Mezzo-Vico Vira, Switzerland	SmartBone®	https://www.ibi-sa.com/products/smartbone/# (accessed date: 09 June 2021)
	Cerapedics, Westminster, CO, USA	i-FACTOR®	https://cerapedics.com/i-factor-and-p-15 (accessed date: 09 June 2021)
